# Conversion between the Brief assessment of Impaired Cognition, Brief Assessment of Impaired Cognition Questionnaire, and Mini-Mental State Examination in a mixed clinical sample

**DOI:** 10.1007/s10072-026-09402-9

**Published:** 2026-09-15

**Authors:** Kasper Jørgensen, T. Rune Nielsen, Daniel Kjaergaard, Frans B. Waldorff, Ann Nielsen, Ane Nørgaard, Lise C. Salem, Thor R. R. Langkilde, Gunhild Waldemar

**Affiliations:** 1 Department of Neurology, Danish Dementia Research Centre, Copenhagen University Hospital - Rigshospitalet, Copenhagen, Denmark; 2 Department of Psychology, University of Copenhagen, Copenhagen, Denmark; 3 Section of General Practice, Department of Public Health, University of Copenhagen, Copenhagen, Denmark; 4 Department of Clinical Medicine, University of Copenhagen, Copenhagen, Denmark

**Keywords:** Assessment, Cognitive impairment, Cognitive screening, Older adults

## Abstract

**Introduction:**

The Brief Assessment of Impaired Cognition (BASIC), and the Brief Assessment of Impaired Cognition Questionnaire (BASIC-Q) are new case-finding tools for cognitive impairment and dementia which are increasingly being used in clinical settings as alternatives to the Mini-Mental State Examination (MMSE). The objective of this study was to develop bi-directional crosswalk tables for conversion between the three instruments for patients 60 years and older.

**Methods:**

Patients from memory clinics and general practice ≥ 60 years of age who completed assessment with BASIC, BASIC-Q, and MMSE were included. The equipercentile equating method with log-linear pre-smoothing of data was applied to create conversion tables between instruments.

**Results:**

A total of 394 patients completed assessment with BASIC and MMSE, 355 of which also completed BASIC-Q. We present bi-directional conversion tables between the three instruments.

**Conclusion:**

The study presents bi-directional crosswalks between BASIC, BASIC-Q and MMSE enabling comparison and synthesis of test results, facilitating communication between clinicians, and potentially helping to inform clinical and policy decision making.

**Supplementary Information:**

The online version contains supplementary material available at https://doi.org/10.1007/s10072-026-09402-9.

## Introduction

Timely identification of cognitive impairment is important for initiating early intervention and care among older adults across health-care settings such as general practice (GP), memory clinics, geriatric wards and outpatient clinics, but also in community health-care services for older adults. Brief cognitive tests and questionnaires are routinely used for case-finding of cognitive impairment and dementia. For the last 50 years the Mini-Mental State Examination (MMSE) has been the most widely used cognitive test for case-finding of dementia. It includes a broad array of tasks and items assessing a wide array of cognitive functioning, except executive function, on a basic level. The MMSE was not specifically developed to identify dementia but aimed at estimating the severity of cognitive impairment among patients in a neurogeriatric ward and monitor cognitive change over time [1]. The MMSE has been extensively validated in multiple clinical populations, including patients with dementia – both any dementia and specific dementia disorders, such as Alzheimer’s disease or vascular dementia – and mild cognitive impairment (MCI). Among the strengths of the MMSE are ease of administration (no certification required), acceptable reliability for a short instrument, and the fact that it has been extensively used for clinical and research purposes for five decades with an estimated 60,000 PubMed-indexed papers citing the original article. Among the limitations of MMSE are its inclusion of heterogeneous factors, ceiling and floor effects, suboptimal sensitivity [2], significant effects of education and age on test performance, and cultural bias [3–6]. Some items, such as the “Serial sevens” subtraction task, may be perceived by patients as confrontational. According to item analysis, half of the items that constitute the MMSE are mainly correlated with age and may be omitted from the test without compromising its diagnostic accuracy [7, 8]. Since 2001, the MMSE has been subject to copyright and licensing restrictions.

The Brief Assessment of Impaired Cognition (BASIC) [9], and the Brief Assessment of Impaired Cognition Questionnaire (BASIC-Q) [10] were developed as case-finding instruments for cognitive impairment and dementia in clinical and community health-care settings. Prior to construction of BASIC and BASIC-Q, preliminary versions of both instruments were tested, and items with low incremental discriminative validity for dementia were excluded based on a series of logistic regression analyses. Both instruments have been validated in memory clinic samples in Denmark and Italy [11–13], in a multicultural memory clinical sample across six European countries [14, 15], and in a GP setting [16, 17]. BASIC has been further validated in nursing home residents in Iran [18], and in stroke patients in Turkey and China [19, 20]. The psychometric properties of BASIC-Q has been examined among community-dwelling older adults in China [21] and its feasibility has been examined in a Danish community setting [22]. BASIC and BASIC-Q are composite instruments [23, 24] combining objective measures of cognitive function with questionnaire items forming the components Self-report, and Informant-report (Table 1, Online Resource Fig. S1).

**Table 1:** The Brief Assessment of Impaired Cognition (BASIC) and the Brief Assessment of Impaired Cognition Questionnaire (BASIC-Q)

Component	BASIC	BASIC-Q
Self-report	Three questions to the patient regarding memory functioning (score 0–6)	
Objective measures of cognitive performance	Supermarket fluency (interval score 0–5)	Orientation in time and own age (score 0–8)
	Category Cued Memory Test (score 0–8)	
Informant-report	Three questions to, e.g., a family member regarding the cognitive functioning of the patient (score 0–6)	
Total score	0–25	0–20

Optimal cutoff score for differentiating between cognitive impairment and normal cognition is 19/20 for BASIC and 16/17 for BASIC-Q

The rationale for including Informant- and Self-report stems from previous research indicating that integration of these types of information with objective cognitive measures may substantially increase the diagnostic accuracy of case-finding instruments [23, 24]. This aligns with current diagnostic criteria for dementia/neurocognitive disorder specifically mentioning that evidence of neurocognitive impairment must be based on “information obtained from the individual, an informant or clinical observation” [25] or “concern of the individual, or a knowledgeable informant, or the clinician” [26]. On a similar note, the Alzheimer’s Association recommend that screening for dementia in primary care should include both objective cognitive assessment and informant-report [27].

The optimal cutoff score for BASIC for identifying cognitive impairment is 19/20 out of 25 points, and 16/17 out of 20 points for BASIC-Q.BASIC performance is significantly correlated with the cognitive status of patients as rated by clinical experts. Education has no significant effect on BASIC or BASIC-Q performance. Age has no significant effect on BASIC-Q and the effect of age on BASIC is numerically too small to justify age adjustment of the cutoff score [9, 10]. BASIC and BASIC-Q were developed from a cross-cultural perspective incorporating items with low cultural bias [28]. The diagnostic accuracy of the instruments does not differ significantly between European native-born and immigrant memory clinic patients, indicating that they are relatively free of cultural bias [14, 15]. BASIC is mainly used as an alternative to the MMSE in GP and other healthcare settings, whereas BASIC-Q is used in community settings and in hospital settings such as geriatric or emergency departments to identify transient or lasting cognitive impairment. BASIC and BASIC-Q can be administered in approximately five minutes each and may be used for clinical, research and other non-commercial purposes without copyright or licensing restrictions. Among the strengths of BASIC and BASIC-Q are the quick and easy administration (also no certification required), acceptable reliability, lack of potentially confrontational items, and good face validity [9, 22].

Numerous studies have presented crosswalk tables for conversion between brief cognitive tests including the MMSE, the Montreal Cognitive Assessment (MoCA) [29], the Rowland Universal Dementia Assessment Scale [30], and Addenbrooke’s Cognitive Examination III (ACE-III) [31]. As BASIC and BASIC-Q are increasingly being applied in a variety of health care settings in Europe and elsewhere there is a need for crosswalk tables for conversion between these instruments and MMSE to enable comparison and synthesis of results, facilitate communication between clinicians, and inform clinical and policy decision making. Such tables may also facilitate monitoring of the cognitive status of patients who are alternately assessed with the instruments over time. Therefore, the aim of this study was to develop bi-directional crosswalk tables for conversion between BASIC, BASIC-Q and MMSE.

## Methods

### Participants

This study combined and retrospectively analyzed data from three sources: (1) a multi-center validation study of BASIC and BASIC-Q in 2018 to 2019 involving memory clinic patients ≥ 65 years of age (*n* = 255), (2) a multi-center validation study of BASIC and BASIC-Q in 2021 to 2022 involving GP patients with cognitive impairment ≥ 70 years of age (*n* = 101), and (3) supplementary data collected in the Copenhagen Memory Clinic in 2025 to 2026 involving patients ≥ 60 years of age with an MMSE score ≤ 20 (*n* = 39) (Table 2).

**Table 2:** Participant characteristics

	Memory clinic, multi-center study (*n* = 255)	General practice (*n* = 100)	Copenhagen Memory Clinic (*n* = 39)
	Mean (SD); range	Mean (SD); range	Mean (SD); range
Age	77.0 (6.42); 65–94 y	79.3 (5.00); 70–92 y	76.4 (7.89); 61–90 y
Sex, male/female	140/115	48/52	12/27
Education (DISCED)	3.2 (1.54); 1–6	3.3 (1.14); 1–6	
BASIC	15.2 (4.27); 3–25	18.0 (4.18); 3–25	11.1 (3.34); 6–19
BASIC-Q	12.3 (4.00); 1–20	14.4 (3.81); 3–20	
MMSE	24.1 (4.45); 8–30	25.0 (3.67); 12–30	15.1 (3.51); 7–20

Abbreviations: *DISCED* Statistics Denmark’s Classification of Education, *BASIC* Brief Assessment of Impaired Cognition (range 0–25), *BASIC-Q* Brief Assessment of Impaired Cognition Questionnaire (range 0–20), *MMSE* Mini-Mental State Examination (range 0–30)

The two multi-center studies have previously been described in detail [9, 10, 16, 17]. In the multi-center memory clinic study, the distribution of clinical diagnoses were Alzheimer’s disease 36%, MCI 16%, vascular dementia 11%, mixed dementia 6%, unspecified dementia 5%, normal pressure hydrocephalus 4%, Lewy body dementia 4%, frontotemporal dementia 4%, alcohol-related dementia 3%, and various neurodegenerative or neurological conditions each contributing with 1–2%, or less. GP patients were classified as cognitively impaired based on a combination of a subnormal performance on the Repeatable Battery for Assessment of Neuropsychological State (RBANS) [32], lack of independence in instrumental activities of daily living according to the Functional Assessment Questionnaire (FAQ) [33], or memory concerns. Patients were eligible for inclusion if they had a close relative or friend who agreed to serve as an informant in the assessment with BASIC and BASIC-Q. Patients were excluded if they were not sufficiently fluent in Danish to participate in assessment without the assistance of an interpreter, or if they had significant sensory deficits invalidating assessment. In the GP validation study patients were classified as cognitively impaired if they had a Total Index score ≤ 75 on the Repeatable Battery for Assessment of Neuropsychological Status (RBANS) [32], and a Functional Assessment Questionnaire (FAQ) [33] score ≥ 6, or reported concerns regarding memory decline [16]. In the two multi-center studies, level of education was categorized into six groups by means of the Danish classification system of educational level, DISCED-15 [34]. The rationale for including supplementary data on memory clinic patients in 2025 to 2026 was to increase the proportion of patients with moderate to severe cognitive impairment in the pooled sample.

### Procedure

As BASIC and BASIC-Q contain overlapping elements in the form of Self-report and Informant-report, they were combined into one instrument during the data collection. In the data collection involving memory clinics, BASIC, BASIC-Q, and the MMSE were administered at the same visit in a random order, and in the collection of data involving GP patients, BASIC and BASIC-Q were administered first, and the MMSE was administered subsequently within one month.

### Statistical analysis

To construct bi-directional crosswalk tables between the instruments, the equipercentile equating method was applied [35]. This is a well-established method for aligning test scores based on their percentile ranks despite a non-linear relationship between the instruments driven by differences in the underlying score distributions within each scale [29]. First, to minimize the effects of sampling error on raw scores these were pre-smoothed by means of iterative Poisson log-linear modelling using Akaike Information Criteria (AIC) and Bayesian Information Criteria (BIC) to evaluate goodness-of-fit and avoid overfitting. Quadratic log-linear models in the form of *f*(x) = *e*^*(a+bx+cx^2*)^ were chosen as the best fit. The addition of a cubic polynomial term did not significantly improve the models. Second, percentile values for the smoothed test scores were calculated and used for aligning scores with roughly equal cumulative percentile ranks. Statistical analyses were performed using R version 4.5.0 with the “equate” package [36], and Microsoft Excel 2016.

## Results

**Fig. 1:**
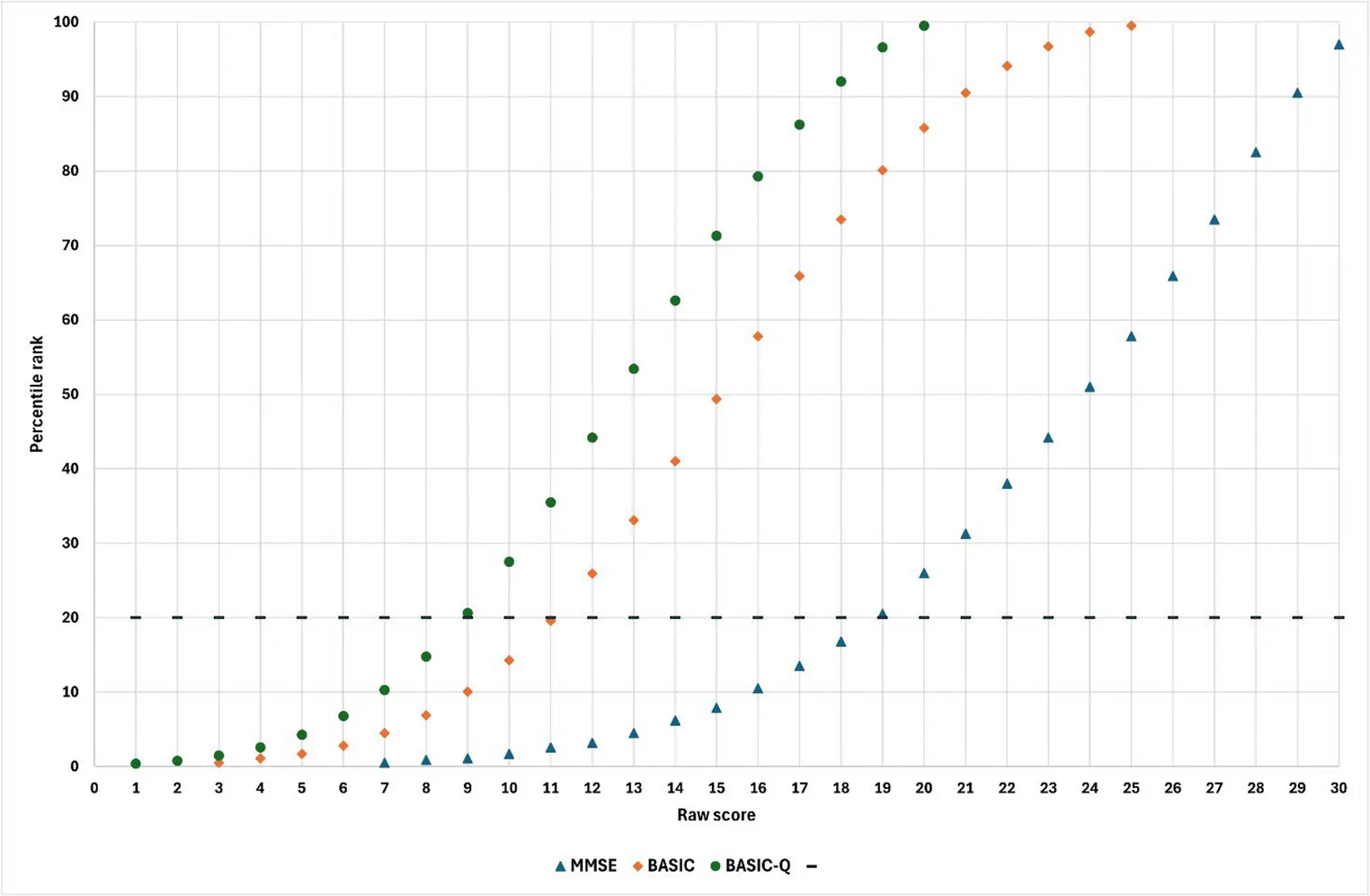
Equipercentile equating of Brief Assessment of Impaired Cognition (BASIC), Brief Assessment of Impaired Cognition Questionnaire (BASIC-Q) and Mini-Mental State Examination (MMSE) raw scores. To illustrate an example of conversion between raw scores, a horizontal reference line representing the 20th percentile has been added. A BASIC-Q raw score of 9 is roughly equal to a BASIC raw score of 11, and to a MMSE raw score of 19. Abbreviations: *BASIC* Brief Assessment of Impaired Cognition; *BASIC-Q* Brief Assessment of Impaired Cognition Questionnaire; *MMSE* Mini-Mental State Examination

The pooled data set contained 394 participants with a mean age of 77.5 years. The participants performed within the score range of 3 to 25 on BASIC, 1 to 20 on BASIC-Q and 7 to 30 on the MMSE. The distribution of raw scores for each instrument and the effects of log-linear pre-smoothing are illustrated in the Online Resources. BASIC scores (Fig. S2) approached a normal distribution whereas MMSE scores were highly left-skewed demonstrating a ceiling effect (Fig. S3) and BASIC-Q scores were moderately left-skewed (Fig. S4). Bi-directional conversions between the three instruments by means of equipercentile equating are presented in Tables 3, 4 and 5, and Fig. 1, and further illustrated in the Online Resources (Fig. S5, Fig. S6, and Fig. S7).

**Table 3:** Bi-directional conversion table for BASIC and MMSE

BASIC	Percentile	MMSE
3–4	*< 1*	7–8
5	*1*	9–10
6	*2*	11–12
7	*3–4*	13
8	*5*	14
9	*7*	15
10	*10*	16–17
11	*15*	18
12	*20*	19
	*25*	20
13	*30*	21
14	*35*	22
	*40*	23
15	*45*	24
16	*50*	
	*55*	25
17	*60*	26
	*65*	
18	*70*	27
19	*75*	
	*80*	28
20	*85*	29
21	*90*	
22–23	*95*	30
24–25	*> 99*	

Abbreviations: *BASIC* Brief Assessment of Impaired Cognition (range 0–25), *MMSE* Mini-Mental State Examination (range 0–30)

**Table 4:** Bi-directional conversion table for BASIC-Q and MMSE

BASIC-Q	Percentile	MMSE
1–2	*< 1*	7
3	*1*	10
4	*2*	12
5	*3–4*	13
6	*5*	14–15
7	*10*	16
8	*12*	17
9	*15*	18
	*20*	19
10	*25*	20
11	*30*	21
	*35*	22
12	*40*	23
13	*45–50*	24
14	*55*	25
	*60*	26
15	*65*	
	*70*	27
16	*75*	
17	*80*	28
	*85*	29
18	*90*	
19	*95*	30
20	*> 99*	

Abbreviations: *BASIC-Q* Brief Assessment of Impaired Cognition Questionnaire (range 0–20), *MMSE* Mini-Mental State Examination (range 0–30)

**Table 5:** Bi-directional conversion table for BASIC-Q and BASIC

BASIC-Q	Percentile	BASIC
1–2	*< 1*	3–4
3	*1*	5
4	*2*	6
5	*3–4*	7
6	*5*	8
7	*10*	10
8	*12–13*	
9	*15*	11
9	*20*	12
10	*25*	
11	*30*	13
	*35*	14
12	*40*	
13	*45*	15
	*50*	16
14	*55*	
	*60*	17
15	*65*	
	*70*	18
16	*75*	19
17	*80*	
	*85*	20
18	*90*	21
19	*95*	23
20	*> 99*	25

Abbreviations: *BASIC-Q* Brief Assessment of Impaired Cognition Questionnaire (range 0–20), *BASIC* Brief Assessment of Impaired Cognition (range 0–25)

Due to the different score distributions and scale lengths, one to one score conversions are not always possible at the high end of the score range. Multiple BASIC scores (22 to 25) correspond to the maximum score of 30 on the MMSE indicating a ceiling effect of the MMSE in this clinical sample. Also, multiple MMSE scores (7 to 12) correspond to scores in the lowest range (3 to 6) on BASIC. Similar patterns are seen in the conversion between BASIC-Q and MMSE.

## Discussion

BASIC and BASIC-Q are increasingly being used as case-finding instruments in a variety of clinical health care and community settings. This study presents bi-directional crosswalk tables for conversion between BASIC, BASIC-Q, and MMSE based on a mixed memory clinic and GP sample with cognitive impairment applying the equipercentile equating method. Other methods for conversion between test scores, such as the circle-arc method, or Rasch analysis, have been reported but are much less frequently used. A few studies have even attempted to use linear regression for test score conversion ignoring the fact that associations between test scores are mostly non-linear. In the present study we also found non-linear associations between BASIC and MMSE, and between BASIC-Q and MMSE, hindering one to one score conversions at both the high and low ranges of the scales.

Although BASIC, BASIC-Q, and MMSE are all used as case-finding instruments for cognitive impairment and dementia and are broadly comparable, it must be emphasized that that the instruments do not necessarily measure the same cognitive domains and that the types of information collected by these instruments are not completely interchangeable. The instruments share some common ground as they contain objective measures of cognitive functioning, but these measures are not identical. BASIC includes a verbal fluency test, assessing language proficiency and executive functioning, and an episodic memory test, but no visuospatial measure. BASIC-Q contains items regarding orientation in time and own age, assessing episodic memory, but no measures of visuospatial or executive function. MMSE contains a wide array of items, including orientation and a memory test, possibly measuring a broader range of cognitive functioning on a basic level, except executive function. In addition to objective cognitive measures, BASIC and BASIC-Q also include Self-report and Informant-report. The inclusion of Self-report can be considered both a strength and a limitation. It contributes significantly to differentiating MCI patients from participants without cognitive impairment, in fact, outperforming the objective components of BASIC and BASIC-Q [11]. But Self-report also makes the instruments susceptible to subjective complaints caused by, e.g., affective or anxiety disorders which generally have a negative impact on cognitive test performance [37, 38]. The proportion of patients with affective or anxiety disorder scoring below the cutoff for cognitive impairment is high on both BASIC (80%) and on BASIC-Q (94%) implying a risk of possible diagnostic misclassification [14]. This problem can be addressed by means of a ratio score comparing objective cognitive performance with subjective complaints (O/S ratio). The O/S ratio can be easily calculated using the components of BASIC: (Supermarket fluency + Memory test)/Self-report, or the components of BASIC-Q: Orientation/Self-report [14, 39]. A high O/S ratio score may be used as a clinical marker to identify patients with possible affective disorder in need of further evaluation, whereas a low ratio score may help to confirm the presence of cognitive impairment.

The diagnostic accuracy of the three instruments involving subsets of the present study population has previously been reported in several studies (Online Resource Table S1). BASIC and BASIC-Q had excellent classification accuracy for dementia and for cognitive impairment (dementia and MCI) in memory clinic settings [9, 10, 12–15], whereas the accuracy in a GP setting may be described as good [16, 17]. To summarize, the classification accuracy of BASIC and BASIC-Q for identification of MCI versus normal cognition was good to excellent [11, 13, 15]. In comparison, the MMSE had good to excellent classification accuracy for dementia and cognitive impairment in both memory clinic and GP settings [9, 16], whereas the classification accuracy for MCI was poor [11]. BASIC is highly accurate in differentiating control participants from both patients with dementia due to Alzheimer’s disease and patients with non-Alzheimer’s dementia (e.g., vascular dementia, Lewy body dementia, or frontotemporal dementia) [12]. The excellent classification accuracy of BASIC and BASIC-Q in memory clinic settings may partly reflect *selection bias* as most patients referred to a memory clinics have significant memory complaints, whereas control participants mostly have no complaints or mild complaints. Both Self-report and Informant-report items specifically focus on memory impairment and contribute with up to 12 points in the total score of BASIC (range 0–25 points) and BASIC-Q (range 0–20 points). Consequently, the probability of being classified as cognitively impaired is increased for patients with subjective memory complaints, or with symptoms of memory impairment as observed by family members, and decreased for participants without such complaints or symptoms.

Among the strengths of this study is the relatively large sample size and the fact that the data set included patients performing across most of the score range of the three instruments covering the spectrum of cognitive impairment for which the instruments are intended to be used. The majority of the sample performed in the mildly impaired or even unimpaired range, but 22% had an MMSE score below 20, and 7% had a score below 15, indicating moderate to severe cognitive impairment.

Among the limitations of the study is the fact that we did not have an independent global measure of the cognitive and functional status (e.g., a Clinical Dementia Rating) of the participants. This makes it more difficult to describe the cognitive status of our sample and compare it with other clinical samples.

Another limitation is the possibility that the validity of the instruments is reduced when assessing patients with moderate to severe cognitive impairment. These patients may have difficulty cooperating on even simple cognitive tasks and their Self-report performance on BASIC and BASIC-Q may be compromised due to a lack of insight and disease recognition. This could potentially compromise the validity of the conversion of scores in the lower end of the scales.

The use of pooled data from memory clinics and GP may be regarded as both a strength and a limitation. On one hand, it makes the results of the study more generalizable, but on the other hand differences in study design and sampling procedures in the two settings may make the results less consistent.

## Conclusion

The presented crosswalk tables between BASIC, BASIC-Q and MMSE enable comparison and synthesis of test results and will hopefully facilitate communication between clinicians in GP, community health-care services, memory clinics, geriatric wards and outpatient clinics and guide clinical decision making. More research is needed to construct crosswalk tables between BASIC, BASIC-Q and other commonly used cognitive instruments.

## Supplementary Information

Below is the link to the electronic supplementary material.

Supplementary Material 1

## Data Availability

The datasets generated during and/or analysed during the current study are available from the corresponding author on reasonable request.
