# Supplementary material for: Conversion between the Brief assessment of Impaired Cognition, Brief Assessment of Impaired Cognition Questionnaire, and Mini-Mental State Examination in a mixed clinical sample
Source: Neurol Sci. 2026 Sep 15;47(10):785. doi: 10.1007/s10072-026-09402-9 (PMC13574889; doi:10.1007/s10072-026-09402-9)
Supplement: Supplementary file 1 [file 10072_2026_9402_MOESM1_ESM.docx]

**Supplementary Material**

**Conversion between Brief Assessment of Impaired Cognition, Brief Assessment of Impaired Cognition Questionnaire, and Mini-Mental State Examination in a mixed clinical sample**

**Supplementary Fig. S1** Brief Assessment of Impaired Cognition (BASIC) and Brief Assessment of Impaired Cognition Questionnaire (BASIC-Q) record forms

**Supplementary Fig. S2** Observed and modelled score frequency for BASIC

Count

|  | Observed frequency | Modelled frequency |
| --- | --- | --- |
| 1 | 0 | 0.324198 |
| 2 | 0 | 0.595949 |
| 3 | 2 | 1.04959 |
| 4 | 1 | 1.7711 |
| 5 | 0 | 2.86337 |
| 6 | 6 | 4.43532 |
| 7 | 5 | 6.5824 |
| 8 | 11 | 9.35958 |
| 9 | 21 | 12.7509 |
| 10 | 18 | 16.6432 |
| 11 | 21 | 20.8135 |
| 12 | 24 | 24.9382 |
| 13 | 24 | 28.6284 |
| 14 | 25 | 31.4878 |
| 15 | 30 | 33.1817 |
| 16 | 39 | 33.5018 |
| 17 | 38 | 32.4078 |
| 18 | 20 | 30.0361 |
| 19 | 25 | 26.6716 |
| 20 | 22 | 22.6917 |
| 21 | 19 | 18.4968 |
| 22 | 23 | 14.4457 |
| 23 | 12 | 10.8092 |
| 24 | 5 | 7.74928 |
| 25 | 3 | 5.3228 |

*N* = 394. Model: *e*^(-1.778+0.673x-(0.0214x^2))

**Supplementary Fig. S3** Observed and modelled score frequency for MMSE

Count

|  | Observed frequency | Modelled frequency |
| --- | --- | --- |
| 1 | 0 | 0.0548639 |
| 2 | 0 | 0.0876328 |
| 3 | 0 | 0.137504 |
| 4 | 0 | 0.211951 |
| 5 | 0 | 0.32094 |
| 6 | 0 | 0.4774 |
| 7 | 1 | 0.697607 |
| 8 | 3 | 1.0014 |
| 9 | 0 | 1.41213 |
| 10 | 1 | 1.95619 |
| 11 | 4 | 2.66206 |
| 12 | 3 | 3.55872 |
| 13 | 6 | 4.67346 |
| 14 | 8 | 6.02911 |
| 15 | 7 | 7.64078 |
| 16 | 8 | 9.51244 |
| 17 | 11 | 11.6336 |
| 18 | 14 | 13.9768 |
| 19 | 20 | 16.4957 |
| 20 | 17 | 19.1251 |
| 21 | 29 | 21.7823 |
| 22 | 16 | 24.3711 |
| 23 | 26 | 26.7866 |
| 24 | 25 | 28.9219 |
| 25 | 31 | 30.6766 |
| 26 | 27 | 31.9637 |
| 27 | 35 | 32.7172 |
| 28 | 39 | 32.8976 |
| 29 | 40 | 32.4954 |
| 30 | 23 | 31.5319 |

*N* = 394. Model: *e*^(-3.389+0.495x-(0.0089x^2))

**Supplementary Fig. S4** Observed and modelled score frequency for BASIC-Q

Count

|  | Observed frequency | Modelled frequency |
| --- | --- | --- |
| 1 | 1 | 0.858731 |
| 2 | 2 | 1.502 |
| 3 | 5 | 2.50753 |
| 4 | 4 | 3.99562 |
| 5 | 8 | 6.07693 |
| 6 | 7 | 8.82157 |
| 7 | 12 | 12.2228 |
| 8 | 14 | 16.1642 |
| 9 | 24 | 20.4034 |
| 10 | 15 | 24.5816 |
| 11 | 17 | 28.2671 |
| 12 | 38 | 31.0252 |
| 13 | 40 | 32.5019 |
| 14 | 35 | 32.4987 |
| 15 | 35 | 31.0159 |
| 16 | 26 | 28.253 |
| 17 | 25 | 24.5644 |
| 18 | 26 | 20.385 |
| 19 | 11 | 16.1464 |
| 20 | 10 | 12.2069 |

*N* = 355. Model: *e*^(-0.758+0.629x-(0.0233x^2))

**Supplementary Fig. S5** Equipercentile conversion between BASIC and MMSE

MMSE score

| BASIC score | MMSE raw score |
| --- | --- |
| 2 | 5 |
| 3 | 7 |
| 4 | 8.5 |
| 5 | 10 |
| 6 | 11.5 |
| 7 | 13 |
| 8 | 14.5 |
| 9 | 16 |
| 10 | 17.5 |
| 11 | 19 |
| 12 | 20 |
| 13 | 21 |
| 14 | 22.5 |
| 15 | 24 |
| 16 | 25 |
| 17 | 26 |
| 18 | 27 |
| 19 | 27.66 |
| 20 | 28.33 |
| 21 | 29 |
| 22 | 29.5 |
| 23 | 29.8 |
| 24 | 30 |
| 25 | 30 |

Abbreviations: *BASIC* Brief Assessment of Impaired Cognition, MMSE: Mini-Mental State Examination

**Supplementary Fig. S6** Equipercentile conversion between BASIC-Q and MMSE

MMSE score

| BASIC-Q score | MMSE raw |
| --- | --- |
| 1 | 6 |
| 2 | 8.5 |
| 3 | 10 |
| 4 | 11.5 |
| 5 | 13 |
| 6 | 14.5 |
| 7 | 16 |
| 8 | 17.5 |
| 9 | 19 |
| 10 | 20.5 |
| 11 | 22 |
| 12 | 23 |
| 13 | 24 |
| 14 | 25 |
| 15 | 26 |
| 16 | 27 |
| 17 | 28 |
| 18 | 29 |
| 19 | 29.5 |
| 20 | 30 |

Abbreviations: *BASIC-Q* Brief Assessment of Impaired Cognition Questionnaire, *MMSE* Mini-Mental State Examination

**Supplementary Fig. S7** Equipercentile conversion between BASIC-Q and BASIC

BASIC score

| BASIC-Q score | BASIC raw |
| --- | --- |
| 1 | 3 |
| 2 | 4 |
| 3 | 5 |
| 4 | 6 |
| 5 | 7 |
| 6 | 8 |
| 7 | 9 |
| 8 | 10 |
| 9 | 11 |
| 10 | 12 |
| 11 | 13 |
| 12 | 14 |
| 13 | 15.5 |
| 14 | 17 |
| 15 | 18 |
| 16 | 19 |
| 17 | 20 |
| 18 | 21.5 |
| 19 | 23 |
| 20 | 25 |

Abbreviations: *BASIC* Brief Assessment of Impaired Cognition, *BASIC-Q* Brief Assessment of Impaired Cognition Questionnaire

**Supplementary Table S1** Diagnostic accuracy results from previous studies

| **Instrument** | **Setting** | **Comparison** | **N** | **Cut-off** | **Sensitivity** | **Specificity** | **AUC** | **Reference** |
| --- | --- | --- | --- | --- | --- | --- | --- | --- |
| BASIC (0-25) | Memory clinic | Cognitive impairment vs normal cognition | 162 vs 109 | 20/21 | 0.95 (0.90-0.98) | 0.95 (0.91-0.98) | 0.98 (0.97-1.00) | [1] |
|  |  | Cognitive impairment vs normal cognition | 309 vs 119 | 19/20 | 0.92 (0.89-0.95) | 0.77 (0.67-0.84) | 0.93 (0.91-0.96) | [2] |
|  |  | Cognitive impairment vs normal cognition | 309 vs 119 | 17/18 | 0.84 (0.79-0.88) | 0.87 (0.80-0.93) | 0.93 (0.91-0.96) | [2] |
|  |  | Cognitive impairment vs normal cognition | 204 vs 42 | 18/19 | 0.91 (0.86-0.95) | 0.86 (0.71-0.95) | 0.96 (0.92-0.98) | [3] |
|  |  | Cognitive impairment vs normal cognition | 204 vs 42 | 19/20 | 0.96 (0.93-0.98) | 0.81 (0.69-0.93) | 0.96 (0.92-0.98) | [3] |
|  |  | Dementia vs normal cognition | 122 vs 109 | 19/20 | 0.95 (0.89-0.98) | 0.98 (0.93-1.00) | 0.99 (0.98-1.00) | [1] |
|  |  | Dementia vs normal cognition | 57 vs 94 | 19/20 | 0.95 (0.84-0.99) | 0.79 (0.69-0.86) | 0.94 (0.91-0.98) | [4] |
|  |  | Dementia vs normal cognition | 116 vs 42 | 15/16 | 0.90 (0.83-0.95) | 0.95 (0.84-0.99) | 0.98 (0.95-1.00) | [3] |
|  |  | AD dementia vs normal cognition | 67 vs 109 | 19/20 | 0.99 (0.91-1.00) | 0.98 (0.93-1.00) | 1.00 (1.00-1.00) | [5] |
|  |  | Non-AD dementia vs normal cognition | 50 vs 109 | 19/20 | 0.90 (0.77-0.96) | 0.98 (0.93-1.00) | 0.99 (0.98-1.00) | [5] |
|  |  | MCI vs normal cognition | 42 vs 83 | 21/22 | 0.86 (0.71-0.94) | 0.89 (0.80-0.95) | 0.95 (0.91-1.00) | [6] |
|  |  | MCI vs normal cognition | 36 vs 94 | 21/22 | 0.94 (0.80-0.99) | 0.64 (0.53-0.73) | 0.86 (0.79-0.92) | [4] |
|  |  | MCI vs normal cognition | 88 vs 42 | 19/20 | 0.92 (0.84-0.97) | 0.81 (0.66-0.91) | 0.92 (0.86-0.97) | [3] |
|  | General practice | Cognitive impairment vs normal cognition | 101 vs 154 | 19/20 | 0.72 (0.62-0.80) | 0.86 (0.79-0.91) | 0.88 (0.84-0.92) | [7] |
|  |  | Mild to moderate cognitive impairment vs normal cognition | 52 vs 154 | 19/20 | 0.85 (0.71-0.93) | 0.86 (0.79-0.91) | 0.92 (0.87-0.97) | [7] |
|  |  | Very mild cognitive impairment vs normal cognition | 49 vs 154 | 19/20 | 0.59 (0.44-0.73) | 0.86 (0.79-0.91) | 0.83 (0.77-0.89) | [7] |
|  | Nursing homes | Cognitive impairment vs normal cognition | 80 vs 120 | 19/20 | 0.91 | 0.90 | 0.96 (0.93-0.99) | [8] |
|  |  |  |  |  |  |  |  |  |
| BASIC-Q (0-20) | Memory clinic | Cognitive impairment vs normal cognition | 159 vs 109 | 16/17 | 0.92 (0.86-0.95) | 0.97 (0.92-0.99) | 0.98 (0.96-0.99) | [9] |
|  |  | Cognitive impairment vs normal cognition | 309 vs 119 | 16/17 | 0.93 (0.89-0.96) | 0.71 (0.62-0.79) | 0.92 (0.89-0.95) | [2] |
|  |  | Cognitive impairment vs normal cognition | 309 vs 119 | 15/16 | 0.87 (0.82-0.91) | 0.81 (0.72-0.88) | 0.92 (0.89-0.95) | [2] |
|  |  | Cognitive impairment vs normal cognition | 204 vs 42 | 15/16 | 0.91 (0.85-0.95) | 0.84 (0.67-0.95) | 0.95 (0.89-0.98) | [3] |
|  |  | Cognitive impairment vs normal cognition | 204 vs 42 | 16/17 | 0.96 (0.92-0.99) | 0.78 (0.64-0.92) | 0.95 (0.89-0.98) | [3] |
|  |  | Dementia vs normal cognition | 57 vs 94 | 14/15 | 0.85 (0.74-0.93) | 0.88 (0.80-0.94) | 0.94 (0.90-0.98) | [4] |
|  |  | Dementia vs normal cognition | 116 vs 42 | 12/13 | 0.83 (0.73-0.91) | 0.97 (0.84-1.00) | 0.97 (0.94-0.99) | [3] |
|  |  | MCI vs normal cognition | 42 vs 83 | 17/18 | 0.88 (0.74-0.96) | 0.88 (0.79-0.94) | 0.94 (0.89-0.99) | [6] |
|  |  | MCI vs normal cognition | 36 vs 94 | 16/17 | 0.83 (0.67-0.93) | 0.73 (0.63-0.82) | 0.82 (0.74-0.90) | [4] |
|  |  | MCI vs normal cognition | 88 vs 42 | 16/17 | 0.95 (0.87-0.99) | 0.78 (0.60-0.91) | 0.91 (0.84-0.97) | [3] |
|  | General practice | Cognitive impairment vs normal cognition | 101 vs 154 | 16/17 | 0.80 (0.72-0.87) | 0.71 (0.63-0.78) | 0.84 (0.79-0.89) | [10] |
|  |  | Mild to moderate cognitive impairment vs normal cognition | 52 vs 154 | 16/17 | 0.85 (0.71-0.93) | 0.71 (0.63-0.78) | 0.87 (0.82-0.93) | [7] |
|  |  | Very mild cognitive impairment vs normal cognition | 49 vs 154 | 16/17 | 0.76 (0.61-0.86) | 0.71 (0.63-0.78) | 0.82 (0.75-0.88) | [7] |
|  |  |  |  |  |  |  |  |  |
| MMSE (0-30) | Memory clinic | Cognitive impairment vs normal cognition | 162 vs 109 | 27/28 | 0.76 (0.68-0.82) | 0.81 (0.72-0.88) | 0.86 (0.81-0.90) | [1] |
|  |  | Dementia vs normal cognition | 122 vs 109 | 26/27 | 0.82 (0.73-0.88) | 0.90 (0.82-0.95) | 0.92 (0.88-0.96) | [1] |
|  |  | MCI vs normal cognition | 42 vs 83 | 28/29 | 0.61 (0.45-0.75) | 0.72 (0.61-0.81) | 0.67 (0.56-0.77) | [6] |
|  | General practice | Cognitive impairment vs normal cognition | 101 vs 154 | 27/28 | 0.71 (0.61-0.80) | 0.87 (0.80-0.92) | 0.85 (0.80-0.90) | [7] |
|  |  | Mild to moderate cognitive impairment vs normal cognition | 52 vs 154 | 27/28 | 0.87 (0.74-0.94) | 0.87 (0.80-0.92) | 0.91 (0.86-0.96) | [7] |
|  |  | Very mild cognitive impairment vs normal cognition | 49 vs 154 | 27/28 | 0.55 (0.40-0.69) | 0.87 (0.80-0.92) | 0.75 (0.67-0.83) | [7] |

Abbreviations: *BASIC* Brief Assessment of Impaired Cognition, *BASIC-Q* Brief Assessment of Impaired Cognition Questionnaire, *MMSE* Mini-Mental State Examination, *AD* Alzheimer’s disease, *MCI* mild cognitive impairment.

1. Jorgensen K, Nielsen TR, Nielsen A, Waldorff FB, Hogh P, Jakobsen S, et al. Brief Assessment of Impaired Cognition (BASIC)-Validation of a new dementia case-finding instrument integrating cognitive assessment with patient and informant report. Int J Geriatr Psychiatry. 2019;34(11):1724-33. doi: 10.1002/gps.5188.

2. Nielsen TR, Jorgensen K, Canevelli M, Pomati S, Delgado-Alvarez A, Franzen S, et al. Validation of the Brief Assessment of Impaired Cognition and Brief Assessment of Impaired Cognition Questionnaire in a multicultural memory clinic sample across six European countries. J Alzheimers Dis. 2025;104(3):823-34. doi: 10.1177/13872877251320259.

3. Canevelli M, Pomati S, Salati E, Maestri G, Sarti G, Calia C, et al. Validation of the brief assessment of impaired cognition (BASIC) and brief assessment of impaired cognition questionnaire (BASIC-Q) in Italy. Neurol Sci. 2026;47(4). doi: 10.1007/s10072-025-08691-w.

4. Nielsen TR, Jorgensen K, Delgado-Alvarez A, Franzen S, Lozano-Ruiz A, Ozden M, et al. Comparison of the diagnostic accuracy of five cross-cultural cognitive screening instruments for dementia and mild cognitive impairment in a multicultural memory clinic sample. J Alzheimers Dis. 2025;106(3):1143-52. doi: 10.1177/13872877251351037.

5. Jorgensen K, Nielsen TR, Nielsen A, Waldorff FB, Hogh P, Gottrup H, et al. Validity of the Brief Assessment of Impaired Cognition case-finding instrument for identification of dementia subgroups and staging of dementia. Eur J Neurol. 2023;30(3):578-86. doi: 10.1111/ene.15636.

6. Jorgensen K, Nielsen TR, Nielsen A, Waldorff FB, Waldemar G. Validation of the Brief Assessment of Impaired Cognition and the Brief Assessment of Impaired Cognition Questionnaire for identification of mild cognitive impairment in a memory clinic setting. Int J Geriatr Psychiatry. 2020;35(8):907-15. doi: 10.1002/gps.5312.

7. Jorgensen K, Nielsen TR, Nielsen A, Oxboll AB, Gerner SD, Waldorff FB, et al. Diagnostic accuracy of the Brief Assessment of Impaired Cognition case-finding instrument in a general practice setting and comparison with other widely used brief cognitive tests-a cross-validation study. Eur J Neurol. 2024;31(10):e16418. doi: 10.1111/ene.16418.

8. Cheraghi M, Seif Panahi MS, Weisi F, Rezaei B, Farhadian M. Translation and validation of the Persian version of the Brief Assessment of Impaired Cognition (P-BASIC) in older adults. Appl Neuropsychol Adult. 2026:1-9. doi: 10.1080/23279095.2026.2628991.

9. Jorgensen K, Nielsen TR, Nielsen A, Waldorff FB, Waldemar G. Brief Assessment of Impaired Cognition Questionnaire (BASIC-Q)-Development and validation of a new tool for identification of cognitive impairment in community settings. Int J Geriatr Psychiatry. 2020;35(7):693-701. doi: 10.1002/gps.5286.

10. Oxboll AB, Jorgensen K, Nielsen TR, Christiansen SD, Nielsen A, Waldorff FB, et al. Diagnostic accuracy of BASIC-Q for detection of cognitive impairment in a primary care setting - a cross-validation study. BMC Geriatr. 2024;24(1):53. doi: 10.1186/s12877-024-04675-1.
